# Additive Manufactured FeCrNi Medium Entropy Alloy Lattice Structure with Excellent Dynamic Mechanical Properties

**DOI:** 10.3390/ma18102173

**Published:** 2025-05-08

**Authors:** Lei Yuan, Zongshu Li, Wentao Liu, Ao Fu, Jian Wang, Yuankui Cao, Bin Liu

**Affiliations:** 1 China North Nuclear Fuel Co., Ltd., Baotou 014035, China; yuanleiwin@163.com (L.Y.); lizongshu0909@163.com (Z.L.); liuwentao0506@163.com (W.L.); 2 CNNC Key Laboratory on Fabrication Technology of Reactor Irradiation Special Fuel Assembly, Baotou 014035, China; 3 State Key Laboratory of Powder Metallurgy, Central South University, Changsha 410083, China; 213307006@csu.edu.cn (J.W.); caoyuankui@csu.edu.cn (Y.C.)

**Keywords:** lightweight design, lattice structure, FeCrNi medium entropy alloy, selective laser melting, dynamic mechanical behavior

## Abstract

Aerospace and marine engineering impose higher requirements on mechanical properties and lightweight design of materials. In this work, combining the high mechanical properties of FeCrNi medium entropy alloy (MEA) and the lightweight advantages of lattice structure, four types of high-performance FeCrNi MEA lattice structures (BCC, BCCZ, FCC, and FCCZ) were prepared by selective laser melting (SLM) technology, and their dynamic mechanical properties were systematically characterized via split Hopkinson pressure bar (SHPB) method. The results demonstrate that the FCCZ FeCrNi MEA lattice structure exhibits superior comprehensive performance among the four lattice structures, achieving the highest specific compressive strength of 59.1 MPa·g^−1^·cm^−3^ and specific energy absorption of 26.3 J/g, significantly outperforming conventional lattice materials including 316L and AlSi10Mg alloys. Furthermore, the finite element simulation and Johnson-Cook (J-C) constitutive model of the dynamic compression process can effectively predict the microstructural evolution and mechanical response of lattice structure, providing critical theoretical guidance for optimizing the design of high-performance lattice structure materials.

## 1. Introduction

The rapid expansion of aerospace and marine engineering has heightened the demand for advanced structural materials, as these industries encounter increasingly complex environments and challenging operational conditions. In the aerospace sector, materials must endure extreme temperatures and harsh atmospheric conditions, while simultaneously withstanding significant mechanical stresses during critical phases of flight, such as takeoff, landing, and high-speed maneuvers [1,2]. Similarly, in marine applications, materials face constant exposure to corrosive seawater, dynamic loads from waves and currents, and potential impacts from debris or vessel collisions [3]. In both domains, there is a pressing need to enhance fuel efficiency and operational performance, which has led to a strong emphasis on lightweight design. Reducing the weight of aircraft can significantly decrease fuel consumption, lower operational costs, and increase payload capacity. Similarly, lighter marine vessels can achieve higher speeds with the same power output and exhibit improved maneuverability. Therefore, the development of lightweight, high-performance structural materials is crucial for promoting the high-quality development of aerospace and marine engineering [4,5].

As a new type of lightweight material, metal lattice structures can effectively meet engineering requirements, exhibiting exceptional specific strength and stiffness that enable significant weight reduction while maintaining high structural stability and load-bearing capacity [6,7]. At present, the materials used for preparing lattice structures mainly include Ti6Al4V [8], AlSi10Mg [9], and 316L alloys [10,11]. The Ti6Al4V and AlSi10Mg lattice structures have the advantages of high strength and low density, but poor plasticity [8,9]. It always breaks at a small deformation, and the buffer energy absorption stage is short. The 316L lattice structure shows good deformation coordination ability and sufficient buffer energy absorption, but the strength and specific energy absorption are still relatively low [10,11]. Therefore, improving the strength of the lattice structure while delaying its failure is the key to improving its impact performance.

Since introduction in 2004, high-entropy alloys (HEAs) have garnered significant attention. HEAs are generally defined as containing five or more principal elements in equimolar or near-equimolar ratios, with a configurational entropy exceeding 1.5 R (where R represents the gas constant). Conversely, those composed of three or four principal elements and with a configurational entropy below 1.5 R are categorized as medium-entropy alloys (MEAs) [12,13,14,15]. Face-centered cubic (FCC) MEAs and HEAs exhibit exceptional strength-ductility synergy and strain hardening capacity due to their unique high-entropy effect, lattice distortion effect, and sluggish diffusion effect, making them promising candidates for lattice structure materials [12,13]. For instance, the CrMnFeCoNi alloy demonstrates excellent mechanical properties at room temperature (tensile strength of 410 MPa and elongation of 57%) [16]. The CoCrNi alloy, owing to its low stacking fault energy, exhibits a high twinning-induced plasticity (TWIP) effect, resulting in exceptional tensile properties at room/low temperatures [17]. Recently, the cobalt-free FeCrNi alloy has garnered significant attention because of its low cost and high mechanical performance [18,19,20]. Selective laser melting (SLM) fabricated FeCrNi alloy exhibits high mechanical properties, with tensile strength and elongation exceeding 1000 MPa and 30%, respectively [21]. Moreover, the FeCrNi alloy shows excellent corrosion resistance due to the high solid-solution concentration of Cr, making it promising for applications in aerospace and marine engineering [22].

Herein, four types of FeCrNi lattice structures (body-centered cubic (BCC), body-centered cubic with *Z*-axis struts (BCCZ), FCC, and FCC with *Z*-axis struts (FCCZ)) were prepared by SLM technology. The dynamic mechanical properties of these lattice structures were evaluated through the split Hopkinson pressure bar (SHPB) method. Additionally, the finite element simulation and Johnson-Cook (J-C) constitutive model were established to predict the microstructural evolution and mechanical response of lattice structure. This work provides guidance for the development of high-performance lightweight lattice structure materials for use in fields such as aerospace and marine engineering.

## 2. Experimental Procedure

**Figure 1:**
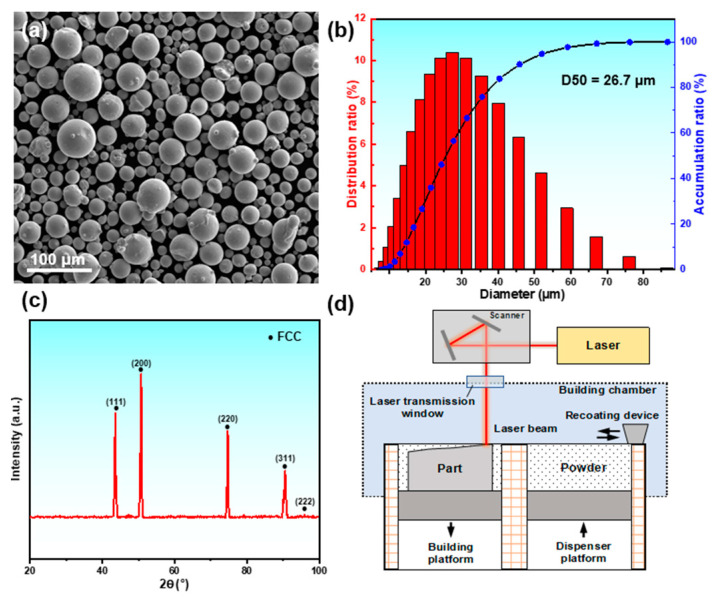
(**a**) The morphology of the pre-alloyed FeCrNi powder and (**b**) the corresponding particle size distribution map. (**c**) The XRD pattern of the pre-alloyed FeCrNi powder showing a single FCC phase. (**d**) The schematic diagram of SLM.

Gas-atomized pre-alloyed FeCrNi powder was used as the raw materials, and the morphology is shown in Figure 1a, exhibiting good sphericity. The particle size distribution was measured by a laser particle size analyzer, and the results is shown in Figure 1b. The particle size of the powder ranges from 8 to 80 μm, with an average particle size of 26.7 μm. The spherical powder with a narrow particle size distribution is suitable for the flow requirement of SLM. The five XRD diffraction peaks in Figure 1c correspond to the (111), (200), (220), (311), and (222) crystal planes of the FCC phase, indicating that the powder has a single-phase FCC structure. The SLM process was carried out on an FS121M machine (Farsoon, Xi’an City, China), and the schematic diagram of SLM is presented in Figure 1d. The SLM parameters were set as follows: laser power of 230 W, scanning speed of 700 mm/s, scanning spacing of 90 μm, layer thickness of 40 μm, and an interlayer rotation angle of 67°. Argon gas was used during the SLM process to maintain the oxygen content within the chamber at below 1000 ppm. To eliminate residual stresses, all specimens were annealed at 400 °C for 3 h and then furnace-cooled.

**Figure 2:**
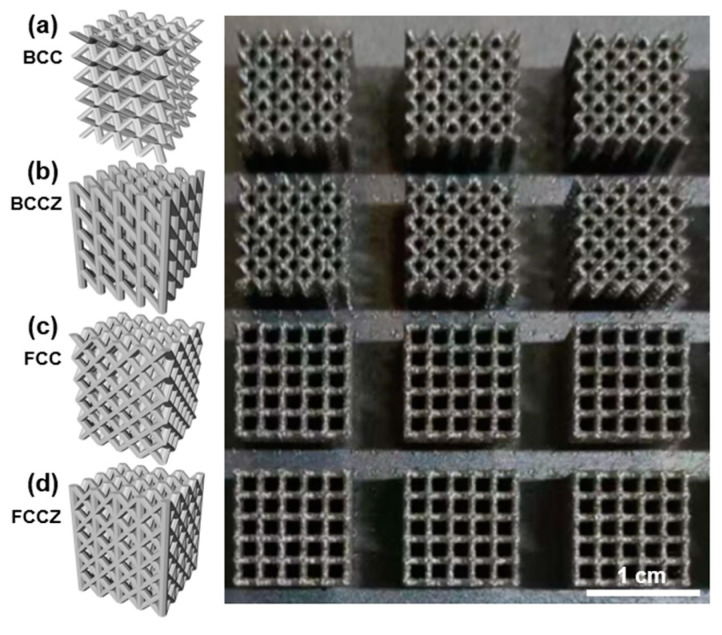
3D models and the printed FeCrNi lattice structures: (**a**) BCC, (**b**) BCCZ, (**c**) FCC, (**d**) FCCZ.

Four types of lattice structures (BCC, BCCZ, FCC, and FCCZ) were selected for 3D printing, and their 3D models were constructed using the 3D Metalwerks software (V1.0). The 3D models, which featured struts with a diameter of 0.3 mm, a unit cell edge length of 2 mm, and a configuration of 5 × 5 × 5 unit cells, are shown in Figure 2a–d along with the corresponding as-printed FeCrNi lattice structures (top-down view). It can be found that the as-printed FeCrNi lattice structures demonstrate high fabrication quality, as evidenced by their structural integrity and high consistency with the designed models, with no observed deformation, fracture, or discontinuity. According to the designed 3D model, the relative densities of BCC, BCCZ, FCC, and FCCZ lattice structures to solid FeCrNi are 35%, 40.7%, 35.9%, and 39.5%, respectively, and the density of FeCrNi MEA is 7.9 g·cm^−3^, thus the densities of BCC, BCCZ, FCC, and FCCZ are 2.77 g·cm^−3^, 3.22 g·cm^−3^, 2.84 g·cm^−3^, and 3.12 g·cm^−3^, respectively.

**Figure 3:**
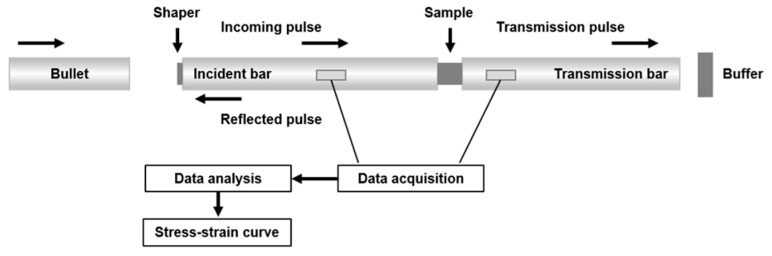
Schematic diagram of the SHPB system. The SHPB test works by generating a stress wave through a striker bar (i.e., bullet) impacting the incident bar, transmitting the wave to the specimen sandwiched between the incident and transmission bars, where reflected and transmitted waves are measured by strain gauges on the bars to calculate dynamic stress-strain behavior.

Phase structure was analyzed by a D/MAX-2250 X-ray diffraction (XRD) machine. Microstructures were performed by an FEI Helios Nanolab G3 scanning electron microscopy (SEM) equipped with an Oxford NordlysMax2 electron backscattered diffraction (EBSD) device, and a Tecnai G2 F20 transmission electron microscopy (TEM). Dynamic compression experiments were tested by a split Hopkinson pressure bar (SHPB) system. The schematic diagram of the SHPB system is shown in Figure 3. Each compression experiment was repeated three times to ensure the stability of the data. Compression simulation analyses were conducted using the ABAQUS finite element analysis software (V6.10). The simulation models were first constructed using the modeling software Spaceclaim, and the generated STP-format files were imported into ABAQUS. Hexahedral meshes were used in the finite element simulation, and in order to ensure simulation accuracy, the meshing of each model consisted of more than 200,000 elements. Von mises yield criterion was applied to simulate the stress distribution during the uniaxial compression process. The upper and lower punches were defined as rigid bodies, and the friction coefficient between the punch and the sample was set to 0.14.

## 3. Results and Discussion

**Figure 4:**
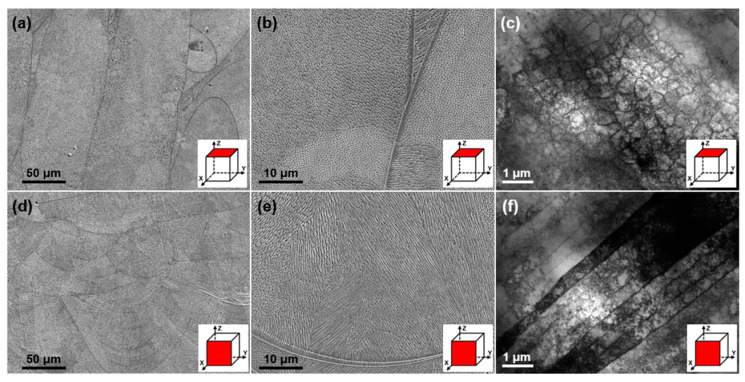
Microstructural characteristics on the XOY plane: (**a**) SEM image showing parallel melt tracks and (**b**) cellular structures with regular pentagon or hexagon morphology; (**c**) TEM bright-field image of the cellular structure composed of high-density dislocations. On the YOZ plane: (**d**) SEM image showing fish-scale-like melt pools and (**e**) cellular structure with elongated columnar morphology; (**f**) TEM bright-field image showing the details of the elongated columnar structure.

The SEM images of the as-printed FeCrNi lattice structure in Figure 4a–d reveal parallel melt tracks on the XOY plane and fish-scale-like melt pools on the YOZ plane, with widths and depths ranging from 70 to 100 μm and 50 to 80 μm, respectively. Both planes exhibit cellular structures commonly observed in additively manufactured alloys, albeit with distinct morphologies. On the XOY plane, the cellular structures appear as regular pentagons or hexagons, whereas on the YOZ plane, they predominantly exhibit elongated columnar morphologies. TEM bright-field images (Figure 4c,f) indicate that the boundaries of these cellular structures are composed of high-density dislocations.

**Figure 5:**
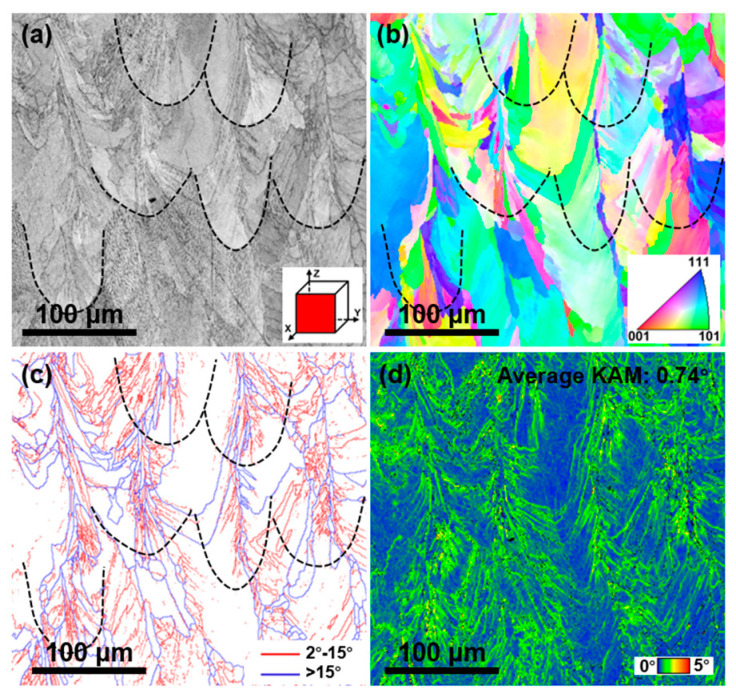
EBSD results of the as-printed FeCrNi lattice structure. (**a**) IQ map showing good image quality of the EBSD sample, where various melt pools can be found. (**b**) IPF map revealing the epitaxial growth of columnar grains across the melt pool. (**c**) GB map showing high-density low-angle grain boundaries (2–15°) within melt pools. (**d**) KAM map with an average KAM value of 0.74°, where higher misorientation corresponds to higher local thermal stress and more GNDs.

Figure 5 shows the EBSD results of the as-printed FeCrNi lattice structure. In the image quality (IQ) (Figure 5a), fish-scale-like melt pools similar to those in Figure 4d are observed. The inverse pole figure (IPF) map (Figure 5b) shows that grains exhibit a coarse, columnar morphology. These columnar grains grow directionally from the melt pool boundary towards the center, aligning with the thermal gradient direction during SLM process. Also, due to layer-by-layer construction, the alloy undergoes repeated heating and cooling. In this thermal history, columnar grains exhibit epitaxial growth across the melt pool. This growth makes grain boundaries less dense at the melt pool boundary than at the center (Figure 5c). Figure 5c also shows a high density of low-angle grain boundaries (2–15°) within melt pools, which are subgrain boundaries from cellular structures. Figure 5d presents the kernel average misorientation (KAM) map, with an average KAM value of 0.74°. The high KAM value indicates high local stress in the as-printed FeCrNi MEA, caused by cyclic heating/cooling during SLM process. In Figure 5d, areas with high misorientation correspond to the high- and low-angle grain boundaries in Figure 5c, especially at the melt pool center, indicating the presence of high-density geometrically necessary dislocations (GNDs) at these locations. These pre-established dislocations enhance alloy performance.

**Figure 6:**
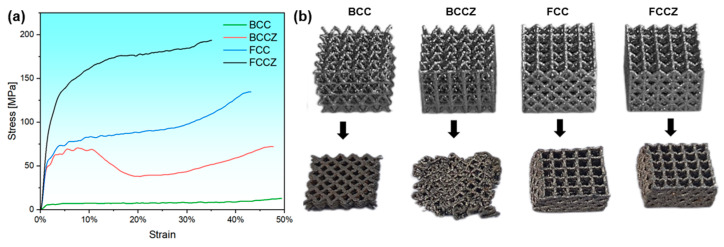
(**a**) Dynamic stress-strain curves of the BCC, BCCZ, FCC, and FCCZ lattice structures. (**b**) Comparison before and after dynamic compression.

Dynamic stress-strain curves of the BCC, BCCZ, FCC, and FCCZ lattice structures are shown in Figure 6a. The plateau stresses (defined as the stable stress after reaching the yield platform) of the BCC, BCCZ, FCC, and FCCZ lattice structures are 8.6 MPa, 68.5 MPa, 94.2 MPa, and 184.5 MPa, respectively, and their specific strengths (defined as the ratio of platform stress to density) are 3.1 MPa·g^−1^·cm^−3^, 21.3 MPa·g^−1^·cm^−3^, 33.2 MPa·g^−1^·cm^−3^, and 59.1 MPa·g^−1^·cm^−3^, respectively. The specific strength of the FCC structure is higher than that of the BCC structure, and the addition of *Z*-axis struts further enhances the strength. The compressive stress-strain curves of the BCC, FCC, and FCCZ lattice structures exhibit an overall upward trend and can be divided into three stages: elastic deformation, plastic deformation, and densification. During the elastic deformation stage, stress increases linearly with strain. In contrast, the compressive stress-strain curve of the BCCZ lattice structure shows a peak stress following elastic deformation, followed by stress fluctuations and a subsequent drop. Upon entering the densification stage, stress gradually increases. The stress drop in the BCCZ lattice structure is attributed to the relatively weak support provided by its inclined struts. Upon impact, the vertical struts undergo significant bending, and the embedded BCC structure within the BCCZ lattice structure fails to deform synergistically with the vertical struts, leading to partial fracture and a reduction in load-bearing capacity along the impact direction. Figure 6b presents a comparison of the four lattice structures before and after dynamic compression. The BCC, FCC, and FCCZ lattice structures retain their original structural integrity after dynamic impact, whereas the BCCZ lattice structure exhibits severe bending and even fracture in some struts, consistent with the characteristics observed in the stress-strain curves shown in Figure 6a.

**Figure 7:**
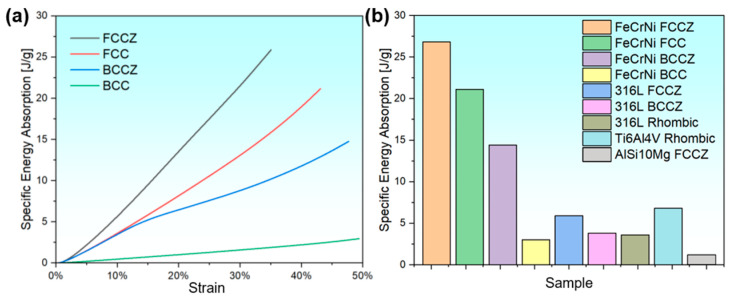
(**a**) Specific energy absorption curves of the BCC, BCCZ, FCC, and FCCZ lattice structures. (**b**) Comparison of specific energy absorption between the FeCrNi lattice structures and other high-performance lattice structures.

Figure 7a presents the specific energy absorption curves of the BCC, BCCZ, FCC, and FCCZ lattice structures. The specific energy absorption is calculated using Formula (1):

Es = E/ρ (1)

where Es is the specific energy absorption, E is the energy absorption, which is calculated by integrating the stress-strain curve in Figure 6, and ρ is the density. During dynamic compression, the specific energy absorption of all four lattice structures increases with increasing strain. The FCCZ lattice structure exhibits the highest specific energy absorption of 26.3 J/g. Except for the BCC lattice structure, the specific energy absorption of the other three lattice structures of the FeCrNi alloy exceeds 13 J/g, significantly outperforming the lattice structures of the 316L, Ti6Al4V, and AlSi10Mg alloys [10,23,24,25], which typically remain below 10 J/g (Figure 7b).

**Figure 8:**
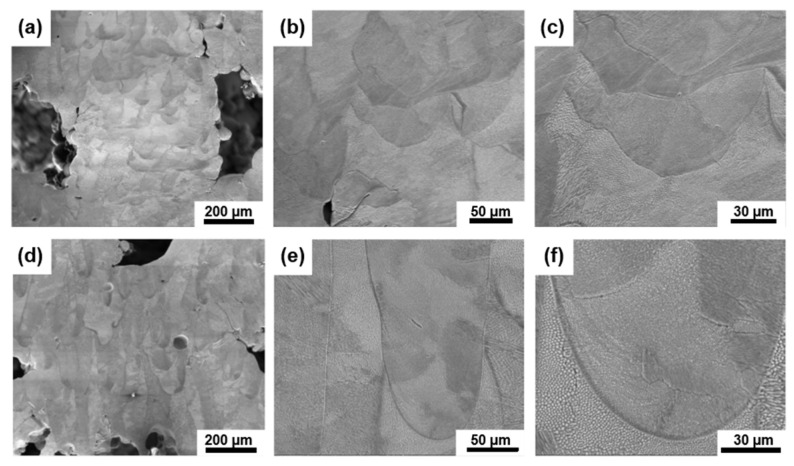
SEM images of the FCCZ lattice structure after dynamic compression at different positions: (**a**–**c**) Vertical strut, and (**d**–**f**) Inclined strut.

The results show that the FCCZ lattice structure exhibits the highest dynamic compressive strength and specific energy absorption. To further analyze its dynamic failure mechanisms, the deformed microstructure of the FCCZ lattice structure was characterized. SEM images of the vertical and inclined struts for the FCCZ lattice structure on the YOZ plane are shown in Figure 8. The height of the melt pools on the vertical struts (Figure 8b,c) is slightly lower than that on the inclined struts (Figure 8e,f), indicating that during dynamic deformation, the vertical struts underwent slightly greater deformation than the inclined struts. Notably, the microstructure after dynamic compression remains similar to that before deformation for both vertical and inclined struts, suggesting that the dynamic deformation of the FCCZ lattice structure is primarily governed by macrostructural deformation, with minimal plastic deformation of the material.

**Figure 9:**
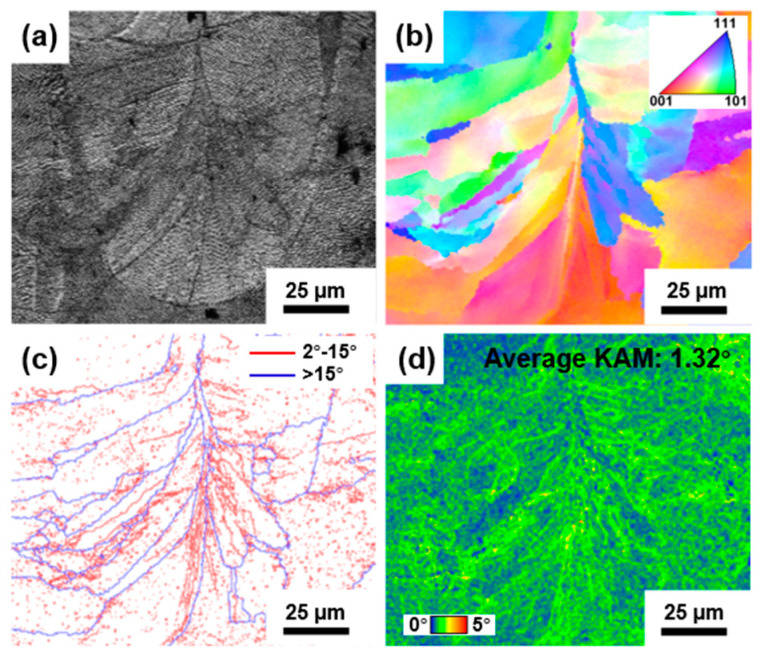
EBSD results of the as-printed FeCrNi lattice structures after dynamic compression (**a**) IQ map showing a single melt pool. (**b**) IPF map showing columnar grains similar to that before deformation. (**c**) GB map revealing the increase of subgrain boundary (2–15°). (**d**) KAM maps with a higher average KAM value of 1.32° indicating a substantial rise in the density of GNDs.

Figure 9 presents the EBSD results of the as-printed FeCrNi lattice structure after dynamic compression in a single melt pool. The IPF map in Figure 9b shows columnar grains similar to those in Figure 5b, with no significant deformation observed. Figure 9c indicates a notable increase in subgrain boundaries compared to Figure 5c. Moreover, the KAM map in Figure 9d shows a significant expansion of regions with high misorientation compared to Figure 5d. The KAM value increased to 1.32°, indicating a substantial rise in the density of GNDs. After impact, typical deformation features of high-entropy alloys, such as deformation twins and deformation bands, were not observed in the microstructure, which suggests that plastic deformation was primarily governed by dislocation movement.

**Figure 10:**
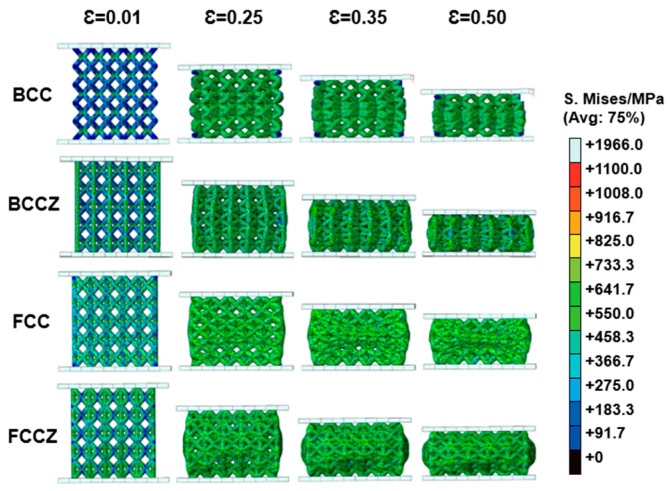
Stress nephograms of the BCC, BCCZ, FCC, and FCCZ lattice structures under compressive strain ε of 0.01, 0.25, 0.35, and 0.50.

The stress nephograms of the BCC, BCCZ, FCC, and FCCZ lattice structures under compressive strain ε of 0.01, 0.25, 0.35 and 0.50 are obtained by finite element simulation (Figure 10). When the strain ε is 0.01, indicating the onset of deformation, the stress concentrations in the BCC lattice structure occurs at the nodes, while in the BCCZ lattice structure, stress concentrations are observed at the nodes between vertical and inclined struts. For the FCC lattice structure, stress concentrations are primarily located on the inclined struts, and in the FCCZ lattice structure, stress is concentrated on both vertical and inclined struts. When stress concentrates at the nodes, the compression process of the lattice structure is dominated by bending deformation. In contrast, when stress concentrates on the struts, the compression process is dominated by axial compressive deformation. Lattice structures with bending-dominated deformation typically exhibit relatively low strength and high compliance, with plateau stress remaining essentially constant during compression [26]. Conversely, lattice structures with axial compression-dominated deformation usually exhibit high strength and cyclic plateau stress after plastic collapse [27]. Therefore, the FCCZ lattice structure, which is dominated by axial compression deformation, exhibits essentially constant plateau stress. The stress responses of the four lattice structures are uniformly distributed throughout the structure and during the deformation process, with the structures collapsing and compacting layer by layer. This explains why the strengths of the BCC, FCC, and FCCZ lattice structures gradually increase in the dynamic stress-strain curves shown in Figure 6a. Despite the anomalous characteristics exhibited by the BCCZ lattice structure in Figure 6a, its specific energy absorption value still increases steadily with increasing strain, consistent with the finite element simulation results.

To better study and predict the flow stress behavior of the FCCZ lattice structure, the J-C constitutive model is employed to describe the dynamic stress-strain curve. Considering the combined effects of strain hardening, adiabatic softening, and strain-rate hardening, the basic form of the J-C model is expressed as [28,29]:

(2) $$\sigma =(A+B\cdot \varepsilon^{n})(1+C\cdot \ln\frac{\dot{\varepsilon }}{\dot{\varepsilon_{0}}})[1-{\left(\frac{T-T_{r}}{T_{m}-T_{r}}\right)}^{m}]$$

where A, B, n, C, and m are material constants; σ is the flow stress; ɛ is the strain; $\dot{\varepsilon }$ is the strain rate; ${\dot{\varepsilon }}_{0}$ = 0.001 s^−1^ is the reference strain rate; T is the temperature, T_m_ = 1400 K is the melting point; T_r_ = 298.15 K is the reference temperature. Thus, the J-C model can be simplified as [30]:

(3) $$\sigma =A+B\cdot \varepsilon^{n}$$

When the strain ε is approximately 0, the value of A is taken as the yield stress under room temperature (298.15 K) and quasi-static conditions (0.001 s^−1^), which is A = 110.2 MPa (Figure S1). By deforming the equation and fitting the curve from the stress (σ) and strain (ε) of the quasi-static stress-strain curve, the parameters k and b are obtained. Here, n = k = 1.2, lnB = b = 5.3, and thus B = $e^{5.3}$ ≈ 193.3 MPa. Therefore, the constitutive equation for the FeCrNi lattice structure at room temperature under quasi-static conditions is:

(4) $$\sigma =110.2+193.3\cdot \varepsilon^{1.2}$$

The function within the second bracket of the J-C model equation represents the strain-rate strengthening parameter. By removing the effects of strain and temperature, the equation can be simplified to focus solely on the strain-rate strengthening effect. With the temperature maintained at room temperature (298.15 K), the value of C can be calculated using the dynamic compression experimental results at different strain rates. When $\dot{\varepsilon }$ ≠ ${\dot{\varepsilon }}_{0}$ and T = T_r_, the equation can be transformed into:

(5) $$C=(\frac{\sigma }{A+B\cdot \varepsilon^{n}}-1)\text{÷}\ln\frac{\dot{\varepsilon }}{\dot{\varepsilon_{0}}}$$

where A = 110.2 MPa, $\dot{\varepsilon }$ is 1100 s^−1^, and ln ($\dot{\varepsilon }$/${\dot{\varepsilon }}_{0}$) is 13.9. By substituting five sets of strain values ε and corresponding flow stress values σ from the plastic deformation stage of the curve, five values of C can be calculated as shown in Table 1. The arithmetic mean of C is determined to be 0.020. Consequently, the J-C constitutive model equation for dynamic deformation flow stress at room temperature can be expressed as:

(6) $$\sigma =(110.2+193.3\cdot \varepsilon^{1.2})(1+0.02\cdot \ln\frac{\dot{\varepsilon }}{\dot{\varepsilon_{0}}})$$

**Figure 11:**
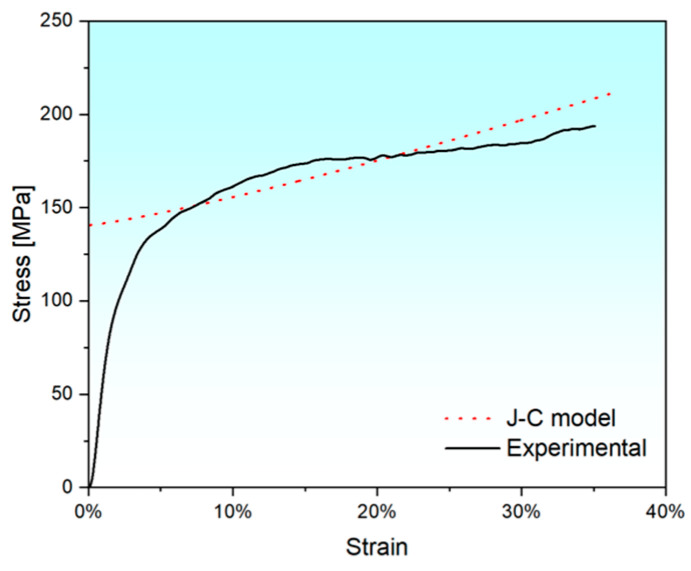
J-C fitting of the FCCZ lattice structure at room temperature and a strain rate of 1100 s^−1^.

The J-C constitutive model is employed to fit the stress-strain curve of the FeCrNi FCCZ lattice structure under dynamic compression at room temperature and a strain rate of 1100 s^−1^. The comparison between the dynamic compressive stress-strain curve of the FCCZ lattice structure and the J-C model is shown in Figure 11. Although the J-C model cannot accurately capture the distinct trends in stress changes during the elastic, plastic, and densification stages of the FCCZ lattice structure at room temperature, the numerical ranges of the two are comparable, and both exhibit an overall upward trend. Therefore, the J-C model can be used for qualitative prediction of the strength of the FCCZ lattice structure under dynamic impact.

## 4. Conclusions

In this study, the FeCrNi lattice structures of BCC, BCCZ, FCC, and FCCZ were successfully fabricated using SLM. Dynamic impact tests were conducted on these lattice structures using SHPB to analyze their dynamic mechanical behavior. The microstructure of the FCCZ lattice structure after dynamic compression was characterized, and finite element simulations combined with J-C model fitting were employed to analyze the dynamic deformation behavior. The following conclusions were drawn:

(1) The FeCrNi lattice structure demonstrates superior specific strength and specific energy absorption compared to other materials like common 316L and AlSi10Mg lattice structures. Its deformation mechanism is primarily governed by macrostructural deformation, with no significant microstructural deformation observed, such as twins and microbands typically seen in MEAs/HEAs.
(2) Among the four lattice structures, the FCCZ structure exhibits the optimal dynamic mechanical performance. Finite element simulations of the compression process reveal that in BCC and BCCZ lattice structures, stress concentrates at the nodes, while in FCC and FCCZ lattice structures, stress concentrates on the struts. The FCC and FCCZ lattice structures, which are dominated by axial compressive deformation, exhibit higher strength. The FCCZ structure, in particular, stands out due to its specific characteristics that enhance its strength and energy absorption capabilities.
(3) The J-C constitutive model for the FeCrNi FCCZ lattice structure at room temperature is expressed as: $\sigma =(110.2+193.3\cdot \varepsilon^{1.2})(1+0.02\cdot \ln\frac{\dot{\varepsilon }}{\dot{\varepsilon_{0}}})$. The J-C constitutive model shows good agreement in predicting the strength and stress response of the FeCrNi FCCZ lattice structure under dynamic impact.

## Figures and Tables

**Table 1:** C value at different ε and σ.

ε	σ	C
0.104	162.8	0.023
0.127	169.1	0.024
0.159	175.6	0.024
0.232	179.4	0.018
0.283	183.6	0.015

## Data Availability

The data presented in this study are available on request from the corresponding author.

## Supplementary Materials

The following supporting information can be downloaded at: https://www.mdpi.com/article/10.3390/ma18102173/s1, Figure S1: Stress-strain curve of the FCCZ lattice structure at the strain rate of 0.001 s^−1^.
