# Supplementary material for: Additive Manufactured FeCrNi Medium Entropy Alloy Lattice Structure with Excellent Dynamic Mechanical Properties
Source: Materials (Basel). 2025 May 8;18(10):2173. doi: 10.3390/ma18102173 (PMC12113326; doi:10.3390/ma18102173)
Supplement: Supplementary file 1 [file materials-18-02173-s001.zip › materials-3606716-supplementary.pdf]

Supplementary Materials

Article

Additive manufactured FeCrNi medium entropy alloy lattice structure with excellent dynamic mechanical properties

Lei Yuan [1], Zongshu Li [1,2], Wentao Liu [1,3], Ao Fu [3,*], Jian Wang [3], Yuankui Cao [3], and Bin Liu [3,*]

[1] China North Nuclear Fuel Co., Ltd., Baotou, 014035, China; yuanleiwin@163.com (L.Y.); lizongshu0909@163.com (Z.L.); liuwentao0506@163.com (W.L.)
[2] CNNC Key Laboratory on Fabrication Technology of Reactor Irradiation Special Fuel Assembly, Baotou, 014035, China; lizongshu0909@163.com (Z.L.)
[3] State Key Laboratory of Powder Metallurgy, Central South University, Changsha, 410083, China; liuwentao0506@163.com (W.L.); aofu_ice@csu.edu.cn (A.F.); 213307006@csu.edu.cn (J.W.); caoyuankui@csu.edu.cn (Y.C.); binliu@csu.edu.cn (B.L.)
* Correspondence: aofu_ice@csu.edu.cn (A.F.); binliu@csu.edu.cn (B.L.)

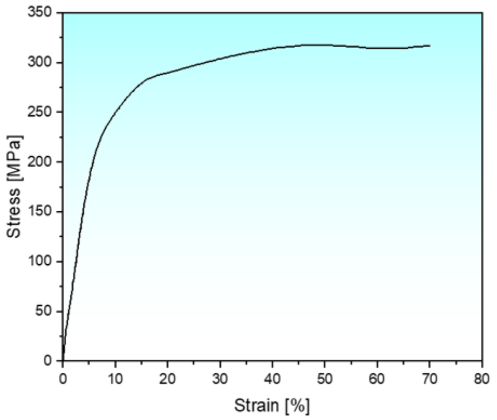

Figure S1. Stress-strain curve of the FCCZ lattice structure at the strain rate of 0.001 s^{-1}.
